# Dopamine increases risky choice while D2 blockade shortens decision time

**DOI:** 10.1007/s00221-022-06501-9

**Published:** 2022-11-09

**Authors:** Stephanie T. Hirschbichler, John C. Rothwell, Sanjay G. Manohar

**Affiliations:** 1 Department of Clinical and Movement Neurosciences, UCL Queen Square Institute of Neurology, London, WC1N 3BG UK; 2 Nuffield Department of Clinical Neurosciences, John Radcliffe Hospital, Oxford, OX3 9DU UK; 3 Department of Neurology, University Hospital St. Pölten, Dunant-Platz 1, 3100 St. Pölten, Austria; 4 Karl Landsteiner University of Health Sciences, Dr. Karl-Dorrek-Straße 30, 3500 Krems, Austria

**Keywords:** Dopamine, Impulsivity, Parkinson disease, Risky decision-making

## Abstract

Dopamine is crucially involved in decision-making and overstimulation within dopaminergic pathways can lead to impulsive behaviour, including a desire to take risks and reduced deliberation before acting. These behavioural changes are side effects of treatment with dopaminergic drugs in Parkinson disease, but their likelihood of occurrence is difficult to predict and may be influenced by the individual’s baseline endogenous dopamine state, and indeed correlate with sensation-seeking personality traits. We here collected data on a standard gambling task in healthy volunteers given either placebo, 2.5 mg of the dopamine antagonist haloperidol or 100/25 mg of the dopamine precursor levodopa in a within-subject design. We found an increase in risky choices on levodopa. Choices were, however, made faster on haloperidol with no effect of levodopa on deliberation time. Shortened deliberation times on haloperidol occurred in low sensation-seekers only, suggesting a correlation between sensation-seeking personality trait and baseline dopamine levels. We hypothesise that levodopa increases risk-taking behaviour via overstimulation at both D1 and D2 receptor level, while a single low dose of haloperidol, as previously reported (Frank and O’Reilly 2006), may block D2 receptors pre- and post-synaptically and may paradoxically lead to higher striatal dopamine acting on remaining striatal D1 receptors, causing speedier decision without influencing risk tolerance. These effects could also fit with a recently proposed computational model of the basal ganglia (Moeller and Bogacz 2019; Moeller et al. 2021). Furthermore, our data suggest that the actual dopaminergic drug effect may be dependent on the individual’s baseline dopamine state, which may influence our therapeutic decision as clinicians in the future.

**Supplementary Information:**

The online version contains supplementary material available at 10.1007/s00221-022-06501-9.

## Introduction

Many neurological and psychiatric diseases such as Parkinson disease or schizophrenia change our decisions, for example, altering preferences for risk or deliberation. These changes are critically influenced by the neurotransmitter dopamine (Assad 2003; Bendiksby and Platt 2006; Ernst et al. 2004; Louie et al. 2011; Malhotra et al. 2013; Maunsell 2004; Small et al. 2003). Risk-seeking involves choosing options that have a high outcome uncertainty, whereas impulsivity relates to choosing fast, without sufficient evidence or deliberation. Clinically, both impulsivity and risk-seeking are linked to treatment with dopaminergic substances (Schaeffer and Berg 2017; Chaudhuri and Schapira 2009). For example, pathological gambling is an impulse control disorder that involves increased risk-taking and is linked to hyperdopaminergic states (Pine et al. 2010; Sinha et al. 2013; Voon et al. 2010; du Hoffmann and Nicola 2014). In line with this, it has been proposed that dopamine controls the effect of risk on choice (Moeller et al. 2021). According to this theory, increasing dopamine levels should lead to risky decision-making. Conversely, dopamine blockade should reduce risky choice.

However, impulsivity and risk-seeking may involve distinct cognitive and neural mechanisms. Decision speed is independently tuned by dopamine levels (Beierholm et al. 2013) via adjustment of decision thresholds (Leventhal et al. 2014; De Corte et al. 2019) but this is not always observed (Nagano-Saito et al. 2012). According to a recently proposed model of the basal ganglia (Opponent Actor Learning model), the value of an option is computed by subtracting its expected losses, encoded in the No-Go pathway, from its expected gains, encoded in the Go pathway (Collins and Frank 2014). Dopamine may control the relative contribution of these two pathways (Mikhael and Bogacz 2016; Moeller et al. 2021):

$$A_{{\text{i}}} = D \times G_{{\text{i}}} - (1 - D) \times N_{{\text{i}}}$$

According to their model, the risk or variance of an option is tracked by the sum of both pathways’ activities (details see Möller and Bogacz 2019), whereas the net expected value is tracked by their difference. As seen in the equation above, the total activation of the action channel (A) depends on the value (G) of the option, minus the risk (N), scaled by dopamine signals (D). This can be viewed as stating that an action’s value is its net expected value plus a risk preference. Hence, simultaneously amplifying both the Go signal (e.g. by stimulating D1 receptors) and the No-Go signal (e.g. by stimulating D2 receptors) increases the estimated value of risky options (Moeller et al. 2021; Moeller and Bogacz 2019). We suggest that the increased activity at both pathways might also speed up decisions, since it ‘energises’ decision-making. Conversely, activation of the D1 pathway while blocking D2 receptors would amplify value (increase Go but not No-Go) signals and thereby also increase decision speed. According to this interpretation, drugs that block D2 receptors might not reduce risky choices, even in those same individuals where elevated dopamine acting on both pathways simultaneously promotes risk-seeking.

In clinical practice, not everybody becomes impulsive on dopaminergic drugs, and the reason for this remains elusive. Amongst other factors, it may also depend on an individual’s endogenous baseline dopamine state (Gjedde et al. 2010) and premorbid tendency towards reward hypersensitivity (Drew et al. 2020). Sensation-seeking is a questionnaire-based trait that indexes the tendency to engage in risky behaviour in daily life and has been correlated with genetic variation at D2 and D4 receptor loci (Ratsma et al. 2001; Hamidovic et al. 2009; Derringer et al. 2010), as well as striatal D2/3 receptor availability, shown using 11C-raclopride PET (Gjedde et al. 2010). Based on this PET evidence, it is argued that high sensation-seekers have both higher D2/D3 receptor density and higher tonic dopamine levels compared to low sensation-seekers. Indeed, sensation-seeking confers sensitivity to D2 agonists in terms of risk-taking (Norbury et al. 2013), indicating that drug effects may segregate according to trait impulsivity (Kanoodi et al. 2021; Froböse et al. 2018; Hofmans et al. 2020).

To further investigate this, we asked 30 healthy volunteers to complete a risky decision-making task on three occasions. Each time they were either given a placebo, a single dose of levodopa (Madopar 100/25 mg) or the dopamine D2 antagonist haloperidol (2.5 mg). The drugs were chosen, as levodopa  and haloperidol both have been shown to alter goal-directed behaviour (Pleger et al. 2009; Symmonds et al. 2013). In addition, Levodopa is the most commonly used drug in the treatment of motor symptoms in PD and its additional effects on other domains are, hence, of special interest. We used a standard gambling task where participants chose between pairs of gambles with different magnitudes of gains, losses and probabilities of winning vs. losing. To our knowledge, no study has yet measured risk-taking on both dopamine and a dopamine antagonist within the same participants.

Our data show increased risk-taking behaviour after a single dose of levodopa and shortened deliberation times for these choices on haloperidol, without causing a change in risk-taking behaviour—an effect seen in low sensation-seekers only.

## Materials and methods

### Subjects and drug manipulation

**Fig. 1:**
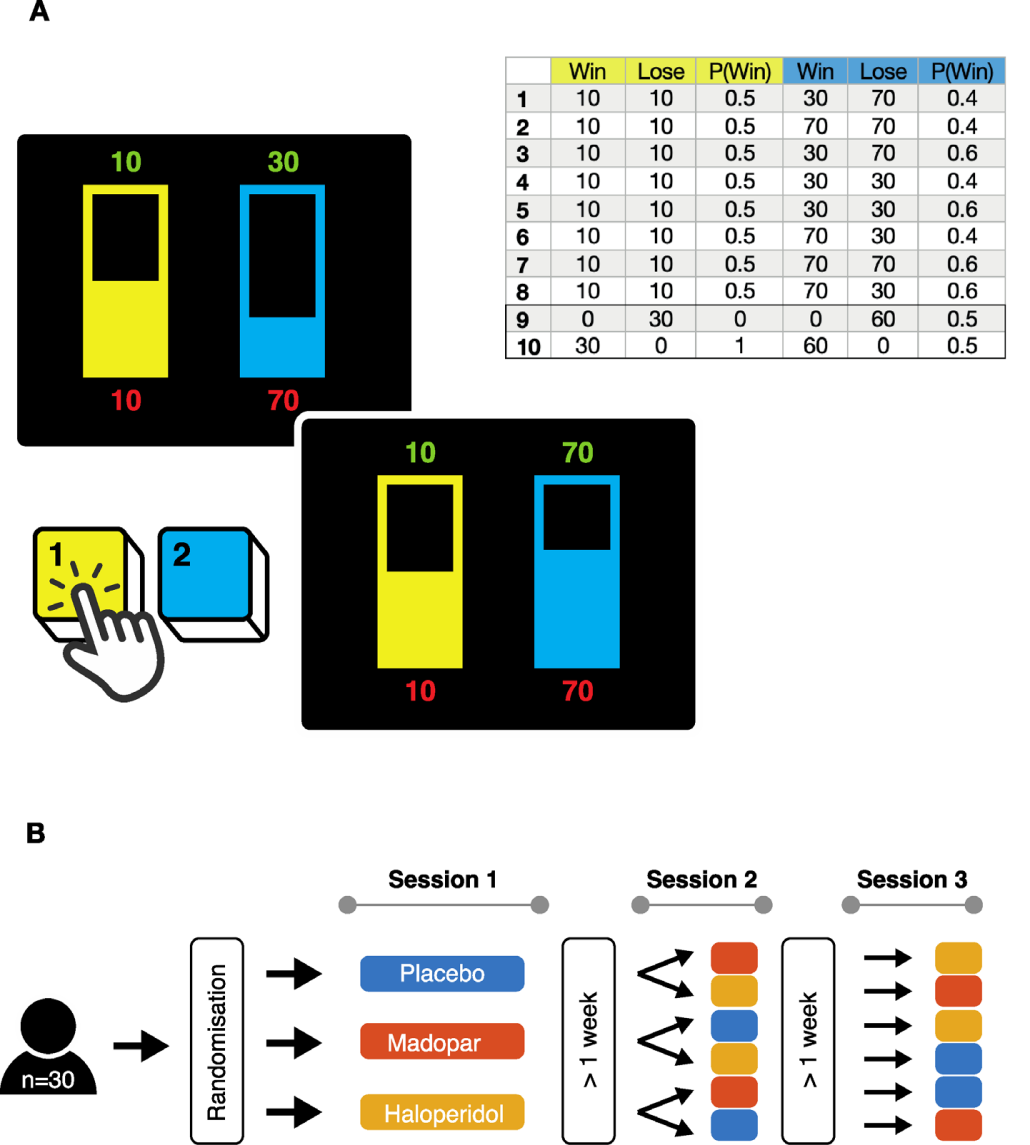
**Gambling task. A** Examples for gambles on offer and a table with all 10 available trial types.** (Top left)** Option of choosing the experimental gamble (displayed in blue by pressing 2 on the keyboard) with a 0.4 probability of winning 30 points and a 0.6 probability of losing 70 points vs. a conservative gamble (displayed in yellow, chosen by pressing 1 on the keyboard) with a 0.5 probability of losing/winning 10 points. **(Bottom) **Example of the option of a 0.6 probability to win 70 points (blue) and a 0.4 probability of to lose 70 points vs. a 0.5 probability of winning/losing 10 points (yellow). The presentation of the two gambles is immediately followed by the choice period, which lasts until the keypress. Following a 1 s delay after the choice was made, feedback of the outcome of the gamble is provided. This is accompanied by two different jingles (one for wins, one for loss outcomes) which are 1 and 2 s long, respectively. After that feedback about the current running total score (1 s) is given. A blank screen is displayed for 1 s subsequently before the next gamble is presented. **(Top right) **Table listing all 10 trial types with amount to lose/win (trials 1–8: experimental = blue; conservative gamble = yellow; certain gain and certain loss trials 9 + 10); *P* (Win) = probability of winning. **B** 30 healthy volunteers were enrolled in the study. After informed consent was given, they were randomly assigned to receive either placebo, Levodopa (100/25 mg Madopar) or haloperidol (2.5 mg) on their first session. After a washout period of at least 1 week, they received one of the remaining two “treatments” for session 2 followed by another washout period before completing session 3 on the last “treatment”. This way there was a total of 6 different drug orders

Thirty healthy volunteers (16 females, mean age: 31.67 ± 12.34 years) attended 3 sessions and were randomly assigned to the order in which they received placebo, levodopa (Madopar 100/25 mg) and haloperidol (2.5 mg). The drug dose of haloperidol was chosen to be small in order to avoid sedative side effects, expecting a potentially mixed effect on pre- and post-synaptical D2 receptors. The participants were blinded for the order of drug administration (Fig. 1B). They had no history of psychiatric, neurological or cardiological illnesses and had not used recreational drugs in the past 3 months. Participants were asked to refrain from drinking or eating 1 h before each session as this may interfere with drug absorption. After taking a BP measurement, participants were given a flavoured drink containing placebo or one of the drugs. This was followed by a waiting period of 1 h after Madopar and placebo and 2 h after haloperidol administration in order to ensure sufficient plasma concentrations. Apart of the data presented here, each session comprised of a number of other behavioural tasks investigating the effect of both drugs on learning, working memory and motivation. Two participants did not complete all three sessions due to time constraints (included datasets placebo: 30, Madopar: 30, haloperidol: 28). The study was approved by the local research ethics committee at University College London and conducted at the UCL Institute of Neurology. All participants gave written informed consent in accordance with the Declaration of Helsinki.

### Task

The task (Rogers et al. 2003; Murphy et al. 2009) comprised of 80 trials and had to be completed on 3 different occasions. On each trial participants were presented with two gambles displayed as two histograms (see Fig. 1). For each option, the possible win was indicated on top of a bar in green numbers, and the possible loss on the bottom in red. The option’s probability of winning was indicated by the height of the colour-filling of the histogram (the higher the filling, the higher the probability to win). On each trial one gamble served as a conservative gamble and had a 50% chance of winning while the second option was an “experimental gamble”, with a variable probability of winning (high 0.6 or low 0.4) and magnitude of wins or losses (large, 70 points vs. small, 30 points). Because of this, the experimental gamble always had a higher risk than the control gamble, as indexed by variability in outcome, but had an expected value that was sometimes higher or lower. Experimental and conservative gambles appeared randomly on the left or the right side of the screen. Participants could choose the left option by pressing “1” on their keyboard and the right one by pressing “2”. This yielded 8 different trial types depending on the combination of sizes of losses/wins and the probability of winning (all trial types see Fig. 1A). In addition, 2 trial types involved a “gains only” and “losses only” option where both gambles available on trial involved either no losses or no gains at all. On *gains only* trials, participants had to choose between a guaranteed win of 30 points and a gamble with a 50:50 probability of winning 60 points or nothing (and vice versa for losses only trials). These 10 trial types were randomised across 4 blocks of each 20 trials. Participants started with a credit of 100 points at the beginning of each block and were presented with the updated credit after they had chosen their respective gamble. Participants were instructed that each gamble should be considered independently of outcomes of previous gambles. They were instructed to make choices that would increase their points score by as much as possible, rather than to respond fast, in line with previous work (Norbury et al. 2013; Rock et al. 2013). A final total was displayed at the end of each block. Mean deliberation times were in line with those observed in previous studies.

In addition, each subject completed the UPPS-P questionnaire to control for individual differences in baseline impulsivity trait and its effect on risk-taking behaviour and interactions between these and drug effects.

### Data handling

We quantified the proportion of trials when the experimental gamble was chosen, as well as the time taken to decide on trials where the experimental gamble was chosen, which we refer to as *deliberation time* in this paper (time between when gambles were displayed and when the choice was made). A mixed linear model was used including the following factors: (1) Effect of drug (using placebo as a reference), (2) magnitude of wins, (3) magnitudes of losses, and (4) probability to win/lose as well as the interaction between drug (1) and each of the three gamble factors (2), (3) and (4). In order to account for different baseline performance between subjects as well as for the two missing datasets, a mixed linear model with random intercept, using the restricted maximum likelihood method, was used and run in R (nlme package). The model fit was assessed using the Akaike Information Criterion (AIC). One analysis per drug using placebo as a reference was run.

In a second analysis, the UPPS-P subscale for sensation-seeking was split according to its median (high vs. low) (as described in Norbury et al 2013) and subsequently added to the model as an additional factor, along with the 2-way interaction with drug effect. No further interactions were included in the model.

## Results

### Madopar increased risk-taking

**Fig. 2:**
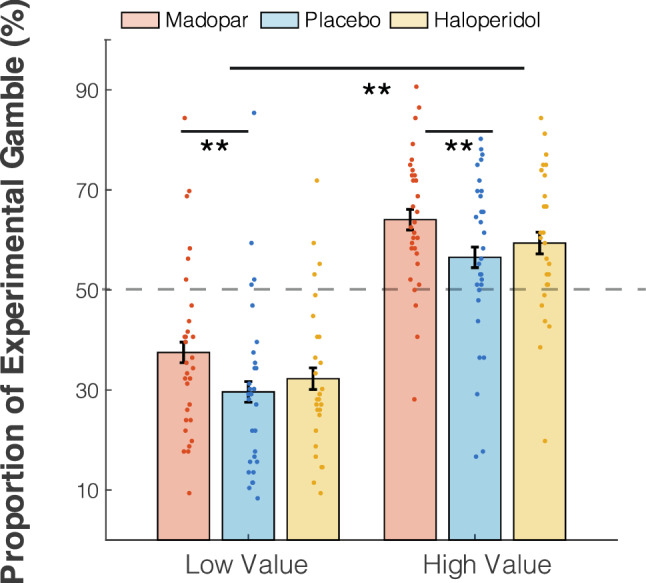
Proportion of experimental gambles—marginal effects (high vs. low expected values)**.** Choices were split into those where the risky gamble was of low expected value (low probability of winning, small wins, high losses) vs. high expected value (high probability of winning, big wins, small losses). High expected value strongly encouraged acceptance of the risky gamble, and drug (Madopar) also promoted risk-taking (**p < 0.001). Error bars indicate within subject errors

As expected, the proportion of experimental gambles was consistently higher when the expected value of the experimental gamble was high in both drug comparisons. In other words, the experimental gamble was chosen more frequently when the size of its wins was large (*p* < 0.001), the size of losses small (*p* < 0.001), and the probability of winning high (*p* < 0.001). Madopar increased participants' tendency to choose the experimental gamble (main effect of drug in the Madopar vs. placebo, *p* < 0.001). There was no main effect of haloperidol on choice (*p* = 0.16), and no interactions between treatment and any of the other factors (*F*-statistics see Table 1, also see suppl. Fig. S1). Figure 2 shows the marginal effect of high- vs. low expected values for the experimental gamble, collapsed across the three gamble variables, for each drug condition.

**Table 1:** Proportion of experimental gambles

Madopar vs. placebo	F	*p*	*β* ± SE
Probability of winning	(1, 443) = 312.17	< 0.001	0.39 ± 0.02
Size of wins	(1, 443) = 82.72	< 0.001	0.20 ± 0.20
Size of loss	(1, 443) = 163.57	< 0.001	0.28 ± 0.02
Drug	(1, 443) = 12.29	< 0.001	0.08 ± 0.02
Prob W * drug	(1, 443) = 0.00	= 1.00	
Size W * drug	(1, 443) = 0.27	= 0.603	
Size L * drug	(1, 443) = 0.06	= 0.813	
Haloperidol vs. placebo			
Probability of winning	(1, 427.23) = 323.57	< 0.001	0.41 ± 0.02
Size of wins	(1, 427.23) = 66.62	< 0.001	0.18 ± 0.02
Size of loss	(1, 427.23) = 138.85	< 0.001	0.27 ± 0.02
Drug	(1, 435.38) = 1.96	= 0.162	
Prob W * drug	(1, 427.23) = 0.583	= 0.445	
Size W * drug	(1, 427.23) = 0.038	= 0.844	
Size L *drug	(1, 427.23) = 0.206	= 0.650	

F-statistics 0 = 0%, 1 = 100% risky choices

### Haloperidol led to faster decisions

**Fig. 3:**
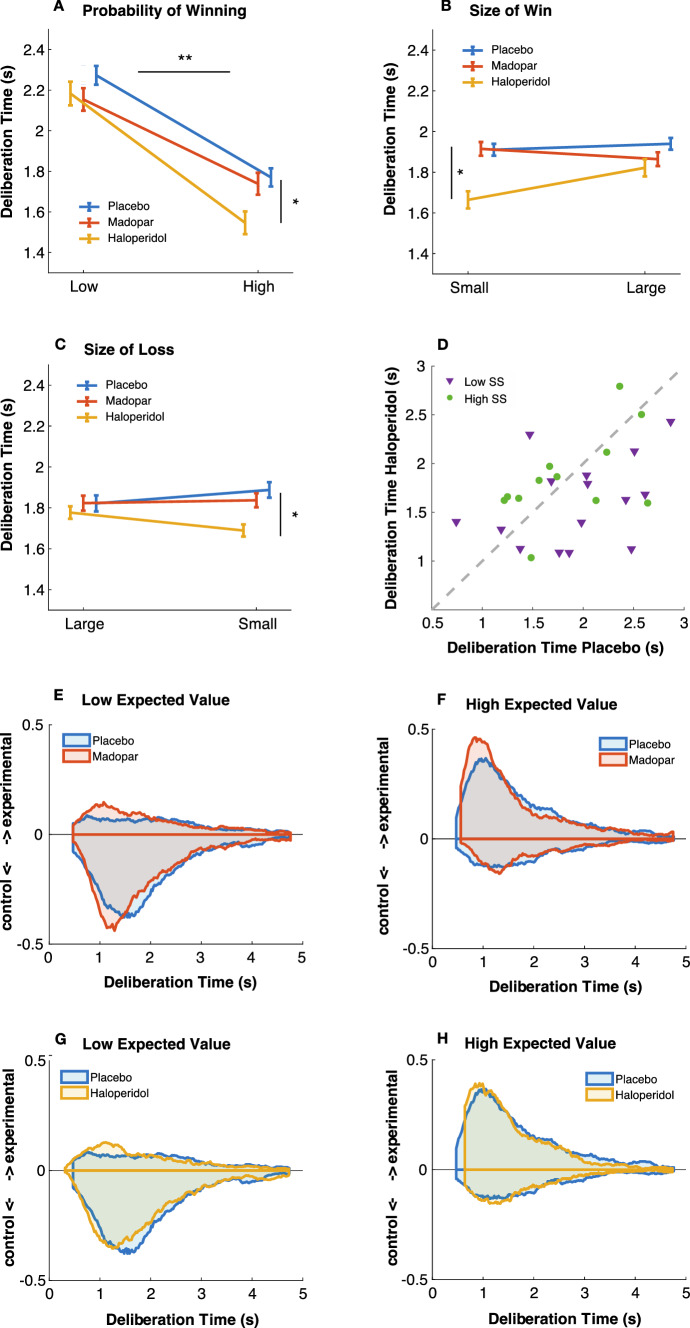
Deliberation time. **A** Deliberation was faster when choosing the option with the higher probability of winning (**p < 0.001). Haloperidol also sped up choices overall (*p = 0.005). **B** There was no effect of size of wins small vs. large. **C** There was no effect of size of losses large vs. small. **D** Individual participant data showing faster deliberation on haloperidol, driven more by the low sensation-seeking participants (SS = sensation-seekers). A similar plot for Madopar vs. placebo for deliberation time as well as the proportion of experimental gambles is provided in supplementary figure S1. **E**–**H** Histograms of deliberation times, showing the timing of choices of the experimental, risky gamble (above the axis) and of the control lower-risk gamble (below the axis). Trials are split into those where the experimental gamble had either a lower expected value (**E**, **G**) or higher expected value (**F**, **H**) than the control gamble

Participants were faster to select the risky gamble when the probability of winning was high in both drug comparisons (*p* < 0.001, Fig. 3A–C). The size of wins or losses did not influence deliberation times. Haloperidol, however, led to faster decisions when compared to placebo (Fig. 3A–C, blue vs. yellow lines, *p* = 0.005), driven mostly by low sensation-seekers (Fig. 3D**)**. Madopar had no effect on deliberation time (main effect of drug in the Madopar vs. placebo comparison, *p* = 0.14, F-statistics see Table 2; Fig. S1). Histograms of the reaction times suggest that this is driven by choices of both the experimental and control gamble (Fig. 3E–H).

**Table 2:** Deliberation time

Madopar vs. placebo	F	*p*	ß ± SE (ms)
Probability of winning	(1, 336.99) = 39.17	< 0.001	− 408.39 ± 65.25
Size of wins	(1, 335.19) = 0.03	= 0.854	
Size of loss	(1, 335.55) = 0.99	= 0.316	
Drug	(1, 335.28) = 2.13	= 0.145	
Prob W * drug	(1, 335.05) = 0.89	= 0.347	
Size W * drug	(1, 334.48) = 0.24	= 0.622	
Size L*drug	(1, 335.20) = 0.02	= 0.884	
Haloperidol vs. placebo			
Probability of winning	(1, 320.98) = 51.09	< 0.001	− 481.13 ± 67.31
Size of wins	(1, 318.22) = 0.37	= 0.545	
Size of loss	(1, 319.00) = 0.24	= 0.625	
Drug	(1, 322.24) = 8.13	= 0.005	− 194.85 ± 68.34
Prob W * drug	(1, 317.77) = 0.69	= 0.407	
Size W * drug	(1, 317.37) = 0.26	= 0.612	
Size L* drug	(1, 317.58) = 1.41	= 0.236	

F-statistics

### No drug effects on matched-value (gain only and loss only) trials

In these trial types, the expected values of the options are matched, and they differ only in risk. Consistent with previously reported findings, participants chose the experimental gamble significantly more often in the win only trials than the loss trials [Madopar: F (1, 87) = 60.23, *p* < 0.001, haloperidol: F (1, 112) = 48.27, *p* < 0.001, *ß* = −0.40 ± 0.08], indicating greater risk-seeking in a reward context. They were also faster in doing so in the gain only trials when compared to loss only [Madopar: F (1, 67.16) = 4.70 *p* = 0.034, *ß* = 1053.61 ms ± 485.97; haloperidol: F (1, 74.70) = 10.56, *p* = 0.034, *ß* = 1124.75 ms ± 346.08]. No significant effect of drug manipulations was found on either of the analyses in the gain only and loss only trials. Neither of the drugs had a significant effect on the proportion of experimental gambles (Madopar: *p* = 0.56, haloperidol *p* = 0.59) nor on the deliberation time (Madopar: *p* = 0.31, haloperidol: *p* = 24).

#### Sensation-seeking increased risk-taking but reduced haloperidol-induced speeding

**Fig. 4:**
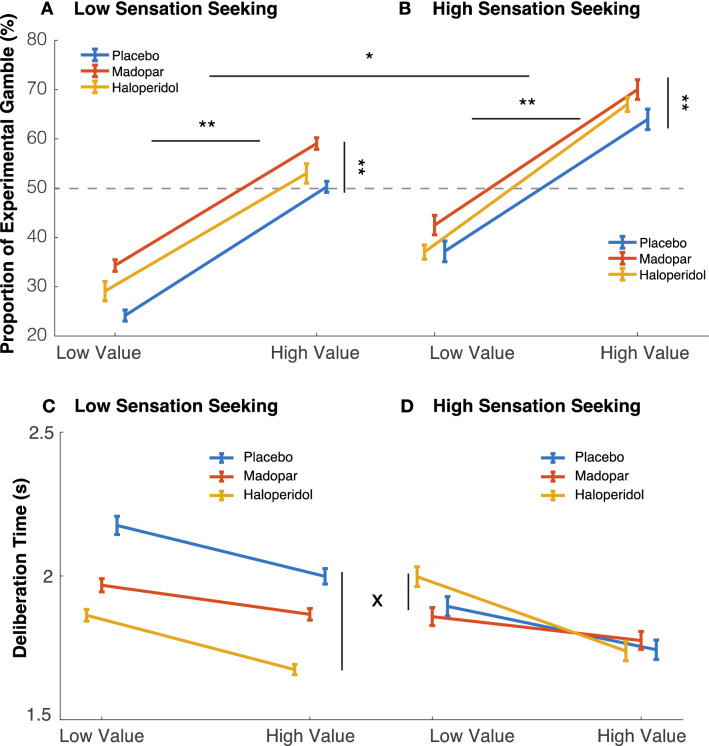
High vs. low UPPS-P sensation-seeking subscale increases gambling risk-taking (**A + B**, *p < 0.05) but reduces effect of haloperidol on deliberation time (**C + D**, x interaction between drug and sensation-seeking trait *p* = 0 0.021). Low sensation-seekers were significantly faster than high sensation-seekers on haloperidol when compared to placebo (blue vs. yellow lines). No significant interaction between Madopar effect and sensation-seeking was found (*p* = 0.96). Marginal effects of low (low probability of winning, small wins, high losses) and high (high probability of winning, big wins, small losses) expected value; Main effect of expected value (**A + B**, **p < 0.001) and drug (Madopar **A + B**, **p < 0.001) on proportion of experimental gambles

Next, we included the UPPS-P subscale for sensation-seeking into the linear model. These models revealed two new significant effects. First, sensation-seeking increased choices of the risky gamble (Fig. 4A + B, main effect of sensation-seeking in both Madopar: F (1, 28) = 5.34, *p* = 0.028, and haloperidol: F (1, 27.58) = 6.42, *p* = 0.017; *ß* = 0.13 ± 0.05). No interaction between sensation seeking and drug was found. (Madopar: *p* = 0.41, haloperidol: *p* = 0.47).

Second, sensation-seeking interacted with haloperidol’s effect on deliberation time (Fig. 4C + D, drug*UPPS-P: F (1, 320.3) = 5.37, *p* = 0.021, *ß* = 301.33 ms ± 129.98). Here, low sensation-seekers sped up when on haloperidol, while no drug effect was observed in the high sensation-seeking group. No interaction between drug and sensation-seeking scores was found in either of the drug comparisons (Madopar vs. placebo: *p* = 0.28; haloperidol vs. placebo: *p* = 0.09).

## Discussion

Weighing risks against benefits in everyday decision-making is crucial for survival and success. Interferences in these processes can lead to apathy, increased risk-seeking behaviour and impulse control disorders, as observed in the context of Parkinson’s disease and treatment with dopaminergic medication, respectively.

Reviewing previous data on pharmacological manipulations in health assessing risk-taking behaviour resulted in somewhat inhomogeneous or even contradictory findings.

While no effect of levodopa on risk-taking was reported (Symmonds et al. 2013), other groups reported increased risk-seeking (Pine et al. 2010) but also reduced risk-seeking behaviour in participants with greater baseline impulsivity scores (Petzold et al. 2019).

The dopamine agonists Pramipexole and Cabergoline were found to promote riskier choices (Riba et al. 2008; Norbury et al. 2015). This was, however, not the case in another study, where Pramipexole showed no effect on risk-taking (Hamidovic et al. 2008), as was the case for bupropion a dopamine re-update inhibitor (Acheson and Wit 2008). Interestingly, indirect dopamine stimulation via a single dose of d-amphetamine even decreased impulsive behaviour in one study (Wit et al. 2002).

In terms of dopamine antagonists Quetiapine promoted risk-seeking in males but not females in the same task used in our study (Rock et al. 2013), although non-dopaminergic mechanisms were suggested to be involved. Pine et al. found a single dose of haloperidol (1.5 mg) not to affect decision time in a delay discounting task (Pine et al. 2010). Possible explanations for these inconsistent findings include factors such as gender, body weight, personality trait (e.g. sensation-seeking) and different drug acting mechanisms (e.g. dopamine release, reuptake, and receptor binding) discussed in Petzold et al. (2019). Another explanatory model is an inverted U-shaped dopamine curve for optimal performance, where extreme highs and lows would hamper optimal decision-making processes (Cools et al. 2003; Rutledge et al. 2015; Petzold et al. 2019).

Here, the idea is that individuals with lower baseline dopamine levels would get impulsive on dopamine antagonistic drugs while those with normal to high dopamine baseline levels would get impulsive on additional dopaminergic stimulation. This theory is supported by data showing tolcapone (a COMT-inhibitor) to decrease impulsiveness in high impulsive participants, when it had no or opposite effects on low impulsive participants (measured by the BIS-11 questionnaire) (Kayser et al. 2012).

In our data, we found two distinct drug effects: **(1)** a single dose of levodopa (Madopar) increased risk-taking behaviour in this risky decision-making task independently of sensation-seeking trait, and **(2)** D2 blockade led to faster deliberation times, an effect that was only seen in low sensation-seekers.

These findings might be best explained by the proposed role of D1 vs. D2 pathways. While the direct/Go pathway may be important for initiating the response, the indirect/No-Go pathway might be important for risk-taking. In the discussed computational model of the basal ganglia (Moeller et al. 2021), dopamine acts at both the Go and No-Go pathway of striatal neurons and amplifies both the expectation of gains (D1-sensitive neurons pathway) and losses (D2-sensitive neurons pathway). The overall effect of stimulating both pathways is to promote the choice of options with greater outcome uncertainty, leading to risk-taking (Moeller and Bogacz 2019; Moeller et al. 2021). This could explain why Madopar increases risk-taking behaviour via overstimulation at both D1 and D2 receptor level. In contrast, amplifying the Go pathway while suppressing the No-Go pathway would in theory make decisions more reward-sensitive, less loss-sensitive, and increase speed. This model could in principle explain our observed effects of haloperidol if a single low dose of haloperidol blocks D2 receptors both pre- and post-synaptically. This would paradoxically lead to higher striatal dopamine acting on remaining striatal D1 receptors, causing faster decisions without influencing risk tolerance.

As deliberation times were shortened in low sensation-seekers, a correlation between D2R-mediated neurotransmission and participants’ sensation-seeking scores is hypothesised. This is in line with a number of human and animal studies (Ratsma et al. 2001; Hamidovic et al. 2009; Kayser et al. 2012) and more specifically with findings from a study using Cabergoline, a D2/D3 receptor agonist in the same task (Norbury et al. 2013). In contrast with the recently proposed theory of an inverted U-shaped relationship between sensation-seeking trait and D2/D3 receptor availability discussed above (Gjedde et al. 2010), Norbury et al. argued, that low sensation-seekers could have a greater “gain” to dopaminergic stimulation compared to participants with higher baseline dopamine levels and, therefore, show stronger effects of dopaminergic stimulation.

Following this rationale, high sensation-seekers should show stronger effects of D2R blockage than low sensation-seekers, which was not the case in our data. It might be possible that in individuals with low baseline D2/D3 receptor availability, eventually a certain threshold may be surpassed, where inhibition of the No-Go pathway leads to speedier responses. This may indicate that inhibitory processes might depend on an “optimal” sweet spot between too much and too little dopamine following an inverted U-shaped curve (Cools et al. 2003; Rutledge et al. 2015).

Although our study has some limitations, e.g., it does not provide direct measures of dopamine, our findings suggest that it may be important to consider individual personality traits, and with this, take individual endogenous baseline dopamine levels into account when choosing study designs.

Further analysing and defining the correlation between drug (side-) effects and baseline dopamine levels is of great importance, as it may help choose tailored treatment strategies for individual patients suffering from PD, optimising treatment efficacy, while avoiding disadvantageous psychiatric side effects.

## Supplementary Information

Below is the link to the electronic supplementary material.

Supplementary file1 (DOCX 1141 KB)

## Data Availability

The data generated and analysed during the current study are available on the OSF repository https://osf.io/9cbsz.
