# Supplementary material for: Dopamine increases risky choice while D2 blockade shortens decision time
Source: Exp Brain Res. 2022 Nov 9;240(12):3351–60. doi: 10.1007/s00221-022-06501-9 (PMC9678996; doi:10.1007/s00221-022-06501-9)
Supplement: Supplementary file 1 — Supplementary file1 (DOCX 1141 KB) [file 221_2022_6501_MOESM1_ESM.docx]

**Figure S1 Relationship between drug effect and sensation seeking scores**

**(A) Deliberation times** were not influenced by Madopar nor sensation seeking scores. **(B)** Haloperidol showed no effect on the proportion of experimental gambles. This was the case independently of sensation seeking trait. **(B)** Madopar increased the proportion of risky choices. No significant interaction with seeking trait was found. **(B-C)** High sensation seekers showed an overall tendency towards riskier choices (Madopar vs. Placebo: p= .028, haloperidol vs. Placebo: p= .017).
